# Phase I clinical trial of the Src inhibitor dasatinib with dacarbazine in metastatic melanoma

**DOI:** 10.1038/bjc.2011.514

**Published:** 2011-11-29

**Authors:** A P Algazi, J S Weber, S C Andrews, P Urbas, P N Munster, R C DeConti, J Hwang, V K Sondak, J L Messina, T McCalmont, A I Daud

**Affiliations:** 1 University of California, San Francisco, MTZ-A741, 1600 Divisadero Street, San Francisco, CA 94143, USA; 2 H Lee Moffitt Cancer Center, Tampa, FL, USA

**Keywords:** melanoma, dasatinib, dacarbazine, Src, biomarkers

## Abstract

**Background:**

Src inhibitors sensitise melanoma cells to chemotherapy in preclinical models. The combination of dasatinib and dacarbazine was tested in a phase I trial in melanoma.

**Methods:**

Patients had ECOG performance status 0–2 and normal organ function. Dacarbazine was administered on day 1 and dasatinib on day 2 through 19 of each 21-day cycle. Both were escalated from 50 mg b.i.d. of dasatinib and 800 mg m^−2^ of dacarbazine. Available pre-treatment biopsies were sequenced for BRAF, NRAS, and C-Kit mutations.

**Results:**

Dose-limiting toxicity was reached at dasatinib 70 mg b.i.d./dacarbazine 1000 mg m^−2^, and was predominantly haematological. In 29 patients receiving dasatinib 70 mg b.i.d., the objective response rate (ORR) was 13.8%, the clinical benefit rate (ORR+SD) was 72.4%, the 6-month progression-free survival (PFS) was 20.7%, and the 12-month overall survival (OS) was 34.5%. Two out of three patients who were wild type for BRAF, NRAS, and c-KIT mutations had confirmed partial responses, and one had a minor response.

**Conclusion:**

The recommended phase II dose is dasatinib70 mg b.i.d with dacarbazine 800 mg m^−2^. PFS and OS data for dasatinib at 70 mg b.i.d. with dacarbazine compared favourably with historical controls. Preliminary data support evaluating tumour mutation status further as a biomarker of response.

The incidence of melanoma is increasing rapidly worldwide. In the United States, an estimated 8700 deaths (Jemal *et al*, 2010) result annually from this disease. The development of metastatic disease is associated with a dismal prognosis (Barth *et al*, 1995) and, until recently, the FDA-approved therapeutic options were not associated with a survival benefit (Atkins *et al*, 1999, 2000; Chapman *et al*, 2011). Ipilimumab, an anti-CTLA-4 antibody, does confer a modest survival benefit in this population, but survival is still limited (median overall survival (OS)=10–11.2 months; Hodi *et al*, 2010; Robert *et al*, 2011). Therapeutic strategies targeting tumour-driving oncogenes now promise to revolutionise the treatment of melanoma. In particular, the BRAF inhibitors vemurafenib and GSK2118643 show evidence of clinical activity in a large proportion of patients whose tumours harbour BRAF^V600E/K^ mutations (Flaherty *et al*, 2010; Kefford *et al*, 2010; Chapman *et al*, 2011). However, about half of the cutaneous melanoma tumours do not harbour BRAF mutations, and even in patients with these mutations, responses to vemurafenib are transient, lasting a median of 6.7 months (Chapman *et al*, 2011). Therefore, the identification of additional therapeutic targets in melanoma is urgently needed.

Given the role of invasion and metastasis in the clinical progression of melanoma, strategies inhibiting these processes could substantially impact the clinical course of the disease. Src and the related Src family kinases signal through multiple downstream intermediaries including STAT3 (Yu *et al*, 1995), FAK, and *β*-catenin (Irby *et al*, 2005), and Src activation has been implicated in decreased tumour cell adhesion, increased invasiveness, and increased motility (Buettner *et al*, 2008). Src activation has been implicated in the pathogenesis of colon (Irby *et al*, 1999; Kline *et al*, 2008), lung (Song *et al*, 2006), pancreas (Trevino *et al*, 2006), breast (Hiscox *et al*, 2006; Jallal *et al*, 2007; Morgan *et al*, 2009), and prostate cancer (Nam *et al*, 2005; Kotha *et al*, 2006). In uveal melanoma, Src activation has been associated with the MAP kinase pathway activation (Maat *et al*, 2009). Src is also frequently activated in cutaneous melanoma (Niu *et al*, 2002; Homsi *et al*, 2009; Eustace *et al*, 2010), and Src overexpression increases cutaneous melanoma cell proliferation and decreases adhesion (Boukerche *et al*, 2010). Conversely, Src inhibition leads to decreased proliferation and migration in melanoma cell lines (Eustace *et al*, 2008, 2010).

Dasatinib is a multi-targeted small-molecule kinase inhibitor that inhibits Src and c-Kit in low nanomolar range. C-Kit is mutated in approximately 15–20% of acral and mucosal melanomas (Beadling *et al*, 2008; Satzger *et al*, 2008; Torres-Cabala *et al*, 2009), and marked objective tumour responses have been observed in patients with exon 11 and exon 13 c-Kit mutant melanoma treated with dasatinib. In one case, this occurred even after disease progression on imatinib (Woodman *et al*, 2009). In melanoma cell lines that have not been selected for c-Kit mutations, dasatinib decreases cellular proliferation (Eustace *et al*, 2010) and enhances apoptosis (Niu *et al*, 2002), and dasatinib decreases cell migration even in cells in which it has no antiproliferative effect (Eustace *et al*, 2008, 2010). Dasatinib may also inhibit the formation of new melanoma lung metastases *in vivo* (Fraser *et al*, 2010). Dasatinib monotherapy is only modestly active in melanoma patients unselected for c-Kit mutations. In a phase II clinical trial, 36 metastatic melanoma patients were treated with dasatinib dosed at 70–100 mg PO b.i.d. Two partial responses were reported and the 6-month progression-free survival (PFS) rate was 13% (Kluger *et al*, 2011). One responding patient had a confirmed c-Kit mutation in exon 13; the other was a c-KIT wild type. Four c-KIT wild-type patients were described with prolonged stabilisation of disease lasting up to 136 weeks. Common dose-limiting toxicities associated with dasatinib in this trial included pleural effusions, dyspnoea, fatigue, and diarrhoea.

In addition to its single agent activity, cell-culture experiments have demonstrated an antiproliferative synergy between dasatinib and chemotherapeutic agents including cisplatin (Homsi *et al*, 2009) and temozolomide (Eustace *et al*, 2008) in c-Kit wild-type melanoma. Dacarbazine is a commonly employed alkylating agent with single-agent activity in advanced melanoma (Luikart *et al*, 1984; Chapman *et al*, 1999; Middleton *et al*, 2000; Schadendorf *et al*, 2006). We conducted a phase I clinical trial of dasatinib in combination with dacarbazine, to determine the safety and tolerability of this regimen in patients with advanced melanoma and to identify a recommended phase II dose. Selective dose expansion cohorts were evaluated for preliminary evidence of efficacy. Tumours from a subset of patients were also evaluated for BRAF, NRAS, and c-Kit mutations as a potential biomarker of response.

## Materials and methods

### Study design

This study was initially designed as a two-part study, with dose-escalation and dose-expansion cohorts. The first three of these cohorts included twice-daily dosing of dasatinib, and the remaining two cohorts included once-daily dosing. The dose escalation for the twice-daily dasatinib dosing schedule was based on a standard phase I dose escalation (3+3) scheme (Ratain *et al*, 1993). Dose expansion was eventually carried out in multiple cohorts for evaluation of dose-limiting toxicity (DLT) events and for better estimation of efficacy and toxicity. A total of five cohorts were evaluated to identify the maximum tolerated dose (MTD) of dacarbazine in combination with dasatinib (Bristol-Myers Squibb, New York, NY, USA). Dosing was as follows: cohort 1, dasatinib 50 mg PO b.i.d, dacarbazine 800 mg m^−2^; cohort 2, dasatinib 70 mg PO b.i.d, dacarbazine 800 mg m^−2^; cohort 3, dasatinib 70 mg PO b.i.d, dacarbazine 1000 mg m^−2^; cohort 4, dasatinib 140 mg PO daily, dacarbazine 1000 mg m^−2^; and cohort 5, dasatinib 100 mg PO daily, dacarbazine 1000 mg m^−2^. Written informed consent was obtained from all patients before the initiation of pre-treatment screening procedures. The Scientific Review Committees and the Institutional Review Boards at the Moffitt Cancer Center in Tampa, FL, USA, and at the University of California, San Francisco, CA, USA, approved the trial. Accrual began in January 2008 and was completed in August 2009.

### Patients

Eligible patients had unresectable stage III or stage IV melanoma. Patients were required to have measurable or evaluable disease, and prior treatment with dacarbazine or temozolomide was not allowed. All patients were required to have an ECOG performance status of 0–2, adequate bone marrow function (absolute neutrophil count >1.5 × 10^9^ l^−1^, haemoglobin >9 g dl^−1^, platelets >100 × 10^9^ l^−1^), renalfunction (creatinine <1.5 times the upper limit of normal), and hepatic function (total bilirubin <1.5 times the upper limit of normal, AST and ALT <2.5 times the upper limit of normal). Patients with vascular events within 6 months were excluded, as were patients with QTc prolongation (>470 ms) and ongoing cardiac dysrhythmias. Patients with leptomeningeal disease were excluded, but neurologically intact patients with treated brain metastasis that had no evidence of recurrence for at least 12 weeks were eligible.

### Study treatment

All patients were treated with dacarbazine on day 1 of each 21-day cycle, and dasatinib was taken orally on treatment days 2 through 19. Dasatinib was not given on treatment days 20, 21, and 1, due to concern that it could decrease cellular proliferation and diminish the efficacy of cytotoxic chemotherapy. Concurrent use of potent CYP3A4 inhibitors or of medications known to prolong the QT interval was prohibited. Before each dacarbazine infusion, patients were pre-medicated with dexamethasone, ondansetron, and lorazepam. Treatment was continued until disease progression, withdrawal of consent, or the development of unacceptable treatment-related toxicity.

### Toxicity assessment

Pre-treatment assessments included a complete history and physical examination, laboratories including a complete blood count, a comprehensive metabolic panel, and a 12-lead electrocardiogram. Adverse events were graded using the Common Terminology Criteria for Adverse Events version 3.0 at weekly follow-up visits during the first 21-day treatment cycle, and every three weeks thereafter. Dose-limiting toxicities were defined as any grade 4 haematological toxicity (except asymptomatic grade 4 neutropenia lasting 7 days or less); prolonged grade 3 or 4 thrombocytopenia (>7 days) or thrombocytopenia associated with bleeding, requiring platelet transfusion; any grade 3 or 4 non-haematological toxicity, despite optimal supportive care; and any toxicity considered unacceptable by the study principal investigator. The MTD was initially defined as the highest dose level at which fewer than two out of six patients (<33%) experienced a DLT in cycle 1, but post-DLT period events were ultimately taken into consideration for defining the MTD. After the 21-day DLT period, dose modifications were made for febrile neutropenia (*T*>38.5°, ANC <1000 mm^−3^); grade 3 or 4 thrombocytopenia, persistent neutropenia (ANC <1000 mm^−3^) or thrombocytopenia (platelet count <100 000 *μ*l^−1^) on day 1 of any treatment cycle; grade 2 or higher liver function abnormalities; diarrhoea; and any other grade 3 or higher non-haematological toxicity other than fever, chills, and flu-like symptoms.

### Response assessment

Planned secondary endpoints included overall response rate (ORR) by the Response Evaluation Criteria in Solid Tumours (RECIST v1.0), OS and PFS. Patients receiving at least two cycles of treatment and those taken off study, early for clinical disease progression were considered evaluable for response. Tumour assessments were made at baseline and at the end of every second cycle (i.e. every 6 weeks). Partial and complete responses were defined by the best treatment response achieved. Stable disease was defined as maintenance of the sum of lesions diameters between a 30% reduction and a 20% increase of overall tumour size over 12 weeks or longer. Patients were assessed for adverse events on the basis of the clinical and laboratory data on treatment days 1, 8, and 15 of the first 21-day cycle, then every 3 weeks on day 1 of each subsequent cycle.

### Statistics

Descriptive Kaplan–Meier survival analysis was performed and subgroup survival comparisons using log-rank were performed using SPSS version 17 (Chicago, IL, USA).

### Exploratory biomarker analysis

A *post-hoc* analysis of tumour mutation status as a predictor of response was performed on the 12 of 46 evaluable patients who had tissue available for analysis. Available pre-treatment, formalin-fixed, paraffin-embedded specimens were used to prepare 5-*μ*m sections. Sections were microdissected to minimise stromal contamination and were genotyped using the PCR for known oncogenic mutations in BRAF exon 15, NRAS exons 1 and 2, c-Kit exons 9, 11, 13, 17, and 18, GNAQ exon 5, and GNA11 exon 5. The two patients with tumours positive for BRAF mutations were identified through screening for a subsequent clinical trial of the BRAF inhibitor PLX-4032 (NCT00949702).

## Results

### Patients and treatment

A total of 51 patients were consented and screened for this study. One patient was found to be ineligible. Another patient entered the study with a diagnosis of melanoma of the soft parts, a disease that is now considered a sarcoma subtype. The patient was included in toxicity, but not response assessments. A total of 49 patients with melanoma, who received any treatment on study, were also considered evaluable for toxicity. Another 46 patients were considered to be evaluable for response. Two inevaluable melanoma patients did not complete two cycles of therapy due to toxicity (*N*=2), and one withdrew consent (*N*=1) and did not have restaging scans. Complete patient demographics are listed in Table 1.

### Toxicity and DLTs

The most common grade 3 and 4 adverse events were haematological (50% of patients overall), including neutropenia, anaemia, and thrombocytopenia (Tables 2 and 3). No DLTs were observed in cohorts 1 and 2. One of the first three patients in cohort 3 (dacarbazine 1000 mg m^−2^, dasatinib 70 mg PO b.i.d) developed dose-limiting thrombocytopenia, and the cohort was expanded to 6 and then to 14 patients to gain additional information regarding treatment-associated toxicity. The sample sizes for cohort expansions were based on the observation of latent (post-DLT period) toxicities rather than pre-planned statistical considerations. Two additional patients in the expansion of cohort 3 developed dose-limiting febrile neutropenia. After the DLT period, one patient developed refractory grade 3 diarrhoea, and another patient had a myocardial infarction that was considered possibly related to treatment. The latent toxicities observed in expansion cohort 3 precluded further clinical development at these dose levels, and dacarbazine 800 mg m^−2^ with dasatinib 70 mg PO b.i.d was designated as the MTD for twice-daily dosing of dasatinib. Cohort 2 was subsequently expanded to a total of 19 patients. This regimen was generally well tolerated, with 9 of 19 patients (47%) experiencing non-dose-limiting (generally transient) grade 3 and 4 haematological adverse events. Grade 3 and 4 non-hematologic adverse events were limited to one patient with grade 3 dyspnoea, two patients with grade 3 hyponatraemia, and one patient with grade 3 hyperglycaemia.

Two regimens with once-daily dasatinib dosing were also evaluated. One out of three patients in cohort 4 (dacarbazine 1000 mg m^−2^ with dasatinib 140 mg PO daily) developed dose-limiting grade 3 thrombocytopenia and grade 3 dyspnoea during the DLT period, and a second patient developed grade 3 dyspnoea on cycle 2 day 15. On the basis of these events, dacarbazine 1000 mg m^−2^ with dasatinib 140 mg PO daily was thought to be too toxic for further development. There were no DLTs in a total of 11 patients treated in cohort 5 (dacarbazine 1000 mg m^−2^ with dasatinib 100 mg PO daily). Five patients in this cohort had non-dose-limiting grade 3 and 4 haematological toxicities, one patient developed grade 3 fatigue, and one patient had an episode of grade 3 gastrointestinal bleeding that was probably treatment related. Further expansion of cohort 5 was not pursued due to the failure of once-daily dasatinib dosing to induce objective tumour responses in these 11 patients.

### Response and survival

**Figure 1:**
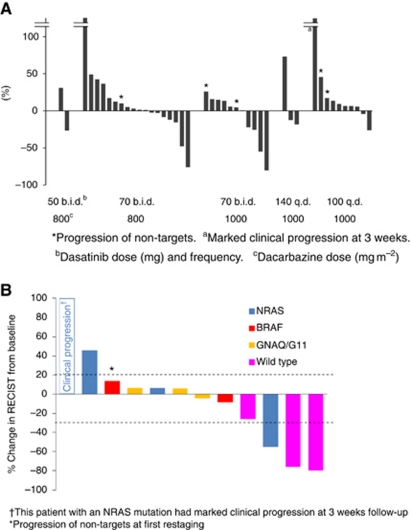
(**A**) Best percent changes in overall RECIST score by treatment group. ^*^Progression of non-targets. ^a^Marked clinical progression at 3 weeks. ^b^Dasatinib dose (mg) and frequency. ^c^Dacarbazine dose (mg m^−2^). (**B**) Exploratory analysis: best RECIST response by tumour mutation status. ^†^This patient with an NRAS mutation had marked clinical progression at 3 weeks follow-up. ^*^Progression of non-targets at first restaging.

A total of 4 out of 29 evaluable patients (13.8%) receiving dasatinib 70 mg PO b.i.d. had partial responses by RECIST (Table 4), and 17 patients had stabilisation of disease for a clinical benefit rate (ORR+SD) of 72.4%. There were no objective responses in the other three cohorts. Prolonged stabilisation of disease was observed in all cohorts and in 26 out of 46 patients (56.5%) overall. Clinical benefit (PR+SD) was observed in 30 of 46 patients (65.2%). The median PFS in all patients was 13.4 weeks and the median OS was 40.6 weeks. The median PFS in cohorts 1–5 was 5.1, 14.0, 13.4, 12.0, and 11.6 weeks, respectively. The median OS in cohorts 1–5 was 63.9, 40.6, 40.9, 28.6, and 40.9 weeks, respectively. In all, 3 out of 12 patients genotyped were wild type for BRAF, NRAS, c-KIT, GNAQ, and GNA11 mutations. Two of these patients had objective tumour responses and one had a minor response (Figure 1). In patients receiving dasatinib 70 mg PO b.i.d., the 6-month PFS rate was 20.7% and the 12-month OS rate was 34.5%.

### Additional subgroup analysis

There were no differences in PFS on the basis of gender, normal *vs* elevated LDH, age, metastatic stage, or ocular *vs* non-ocular primary. Median PFS by genotype was 5.7, 6, 17.3, and 34 weeks for patients with BRAF (*N*=2), NRAS (*N*=4), GNAQ/GNA11 (*N*=3), and wild-type (*N*=3) mutation status, respectively. Patients with clinical benefit from dacarbazine and dasatinib had significantly longer OS than those without (51.6 *vs* 22.3 weeks, *P*<0.01), and there was a trend towards worse OS in patient with ocular *vs* non-ocular primaries (22.9 *vs* 40.9 weeks, n.s.). There were no differences in OS on the basis of gender, age, or metastatic stage, and there were not enough events in the genotyped patients to allow calculation of the median OS in all groups.

## Discussion

The Src family kinases has critical roles in the process of cancer invasion (Kim *et al*, 2009), and preclinical data indicates that Src inhibition may add to tumour control in advanced malignancies including melanoma (Eustace *et al*, 2008; Homsi *et al*, 2009; Fraser *et al*, 2010). In the current study, we show that dasatinib, a Src/abl kinase inhibitor, can be safely combined with dacarbazine at an adequate dose intensity. Dose expansion cohorts at three levels were accrued to profile-delayed toxicities and to get a better estimate of efficacy. Toxicities were primarily haematological, and treatment discontinuations were required with post-cycle 1 therapy in patients receiving dasatinib 70 mg PO b.i.d with dacarbazine 1000 mg m^−2^ IV every 3 weeks. At this dose level, objective responses were noted in 2 out of 11 (18.2%) patients and clinical benefit in 7 out of 11 (54.5%) patients. Treatment was better tolerated in patients receiving 70 mg PO b.i.d with dacarbazine 800 mg m^−2^. Objective tumour responses were observed in 2 patients (11%), and clinical benefit was observed in 11 out of 18 patients (61.1%) in this cohort. On the basis of these findings, the latter dose level would be appropriate for further clinical trials. In contrast to a recent phase II study of dasatinib monotherapy (Kluger *et al*, 2011), grade 3 and 4 pulmonary and gastrointestinal toxicities were infrequent, occurring in only 2 of 33 patients (6.1%) receiving dasatinib 70 mg PO b.i.d. Grade 3 and 4 pleural effusions were not observed. The lower incidence of these side effects in the present study may be due to the steroid premedication used with dacarbazine, dacarbazine itself, or the dasatinib dosing schedule that included a three-day pause in dasatinib dosing on treatment days 20, 21, and 1. Of note, the first 17 patients treated in the prior study started with dasatinib dosed at 100 mg PO b.i.d, and the majority of these patients required early treatment interruptions due to treatment-associated toxicity. The incidence of pulmonary and gastrointestinal toxicity was markedly lower in patients enrolled subsequently at a dose of 70 mg PO b.i.d.

Dasatinib combined with dacarbazine appears to be more active than either agent alone, with the caveat that cross-trial comparisons must be interpreted with extreme caution. In a phase II trial, dasatinib monotherapy yielded a median PFS of 8 weeks and a 6 month PFS rate of 13% (Kluger *et al*, 2011). Similarly, patients treated with dacarbazine alone typically demonstrate objective disease progression on the first restaging scan (e.g. Chapman *et al*, 2011). In contrast, the median PFS for patients treated with dacarbazine+dasatinib was 13.4 weeks and the 6-month PFS rate was 20.7%. Furthermore, both the 6-month PFS and 12-month OS rates for dacarbazine and dasatinib compare favourably with benchmarks established in the Korn *et al* (2008) meta-analysis based on 42 trials, which completed accrual between 1975 and 2005 (6-month PFS=20.7% *vs* 14.5%, 95% CI 12.9–16.1% 12-month OS=34.5% *vs* 25.5%, 95% CI 23.6–27.4%). These findings suggest that the addition of dasatinib to dacarbazine may decrease the dissemination of advanced melanoma lesions, consistent with reductions in tumour cell invasion and migration induced by dasatinib in pre-clinical studies (Eustace *et al*, 2008, 2010).

The current study supports further exploration of BRAF/NRAS/KIT wild-type status as one of several candidate biomarkers that could help to identify patients who are most likely to benefit from dacarbazine with dasatinib. The absence of BRAF and NRAS mutations in melanoma cell lines is associated with a greater sensitivity to dasatinib *in vitro* (Journe *et al*, 2010). Genotyping data from the current study were limited by tissue availability and subject to non-random selection. However, the finding that two of four clinical responders to dasatinib with dacarbazine did not have the BRAF/NRAS/c-Kit mutations is intriguing and bears further evaluation in the context of a phase II clinical trial. Additional candidate biomarkers could also be explored in such a trial including caveolin-1, an Src substrate that influences c-Src kinase activity. Caveolin-1 expression in melanoma is associated with increased cellular proliferation (Felicetti *et al*, 2009; Trimmer *et al*, 2010), and in some studies, enhanced tumour cell invasion and migration (Felicetti *et al*, 2009). Several studies have identified elevated caveolin-1 expression as a biomarker of response to dasatinib in breast, lung, and prostate cancer cells (Finn *et al*, 2007; Huang *et al*, 2007; Wang *et al*, 2007). Furthermore, in a phase II trial of single-agent dasatinib, five out of six metastatic melanoma patients with objective tumour-size reductions had elevated caveolin-1 expression levels before treatment (Jilaveanu *et al*, 2011).

Preliminary data suggest that c-Kit inhibitors, including dasatinib, are highly active in c-Kit mutant melanoma (Lutzky *et al*, 2008; Carvajal *et al*, 2009, 2011; Woodman *et al*, 2009; Handolias *et al*, 2010; Guo *et al*, 2011), and dasatinib is currently in phase II testing in patients with solar, mucosal, and acral melanoma (‘Dasatinib in Advanced Mucosal, Acral, or Solar Melanoma,’ ClinicalTrials.gov, no date). However, in the current study, none of the genotyped patients who benefitted clinically harboured c-Kit mutations.

In summary, the combination of dacarbazine and dasatinib is well tolerated at clinically relevant dose levels. Dacarbazine dose at 800 mg m^−2^ combined with 70-mg dasatinib twice daily warrants further evaluation in the context of a phase II clinical trial in advanced melanoma, and this trial could further evaluate candidate biomarkers for response. These biomarkers, if validated, may help to identify the optimal target population for treatment with dacarbazine and dasatinib in combination.

## Figures and Tables

**Table 1:** Patient demographics

**Patient demographics (*N*=50)**	
Age (years)	62.3±12.1
	
*Gender*	
Male (%)	31 (62)
Female (%)	19 (38)
	
*Stage*	
M1a (%)	6 (12)
M1b (%)	13 (26)
M1c (%)	31 (62)
	
*Prior treatment*	
Chemotherapy (%)	2 (4)
Vaccine (%)	1 (2)
None (%)	47 (94)
	
*Baseline lactate dehydrogenase*	
Normal (%)	32 (64)
Elevated (%)	18 (36)
	
*ECOG performance status*	
0 (%)	23 (46)
1 (%)	22 (46)
2 (%)	2 (4)^a^
	
*Primary site*	
Cutaneous	
Torso (%)	13 (26)
Head and neck (%)	11 (22)
Extremity (%)	11 (22)
Multiple (%)	2 (4)
Unknown (%)	4 (8)
Ocular (%)	6 (12)
Mucosal (%)	2 (4)
Soft parts (%)	1^b^ (2)

a ECOG performance status was not documented for two patients.

b Patient with melanoma of the soft parts included in toxicity, but not response assessments.

**Table 2:** Toxicities possibly, probably, and definitely related to treatment

**Dacarb/dasatinib**	**Grade**	**Heme**	**GI**	**Resp.**	**Integ.**	**Pain**	**Constit.**	**Lab**	**Other**
800/50 b.i.d.	1 and 2	Neutro (1) Anaemia (2) Platelets (1)	Nausea (2) Diarrhoea (2) Anorexia (2) Dehydr (1) Constip (1)		Alopecia (1)	Headache (2)	Fatigue (2)	Low alb (2) Low Na (1) Low Ca (1)	Dizziness (1)
	3 and 4								
800/70 b.i.d.	1 and 2	Neutro (4) Anaemia (14) Platelets (11)	Anorexia (3) Diarrhoea (8) Nausea (13) Constip (5) Dysphagia (1) Dysgeusia (1) Anorexia (2) Mucositis (1)	Effusion (1) Dyspnea (2) Oedema (1)	Rash (7) Alopecia (2) Flushing (3)	Headache (8) Abdomen (1) Myalgias (1) Face (1) Hand (1)	Fatigue (10) Sweats (2) Chills (3) Insomnia (2) Wt loss (1) Fever (4)	High Mg (2) Low Mg (2) Low Ca (1) High gluc (2) Alk phos (5) ALT (5) AST (1) Low alb (2)	Anxiety (1) Oedema (5) Numbness (2) HSR (2) Depress (1) Polyuria (1) Dizziness (1) Neuropathy (1)
	3 and 4	Neutro (6) Anaemia (3) Platelets (3)		Dyspnea (1)				High gluc (1) Low Na (1)	
1000/70 b.i.d.	1 and 2	Neutro (2) Anaemia (6) Platelets (4)	Nausea (11) Diarrhoea (7) Er satiety (1) Constip (1) Anorexia (3) Mucositis (1) Tongue sw (1)	Effusions (3) Atelect (1) Cough (1) Hiccups (1) Dyspnea (1)	Rash (5) Alopecia (3) Pruritis (3) Flushing (1) Dry skin (1)	Foot (1) Headache (4) Myalgias (1) Abdomen (2)	Fatigue (4) Chills (2) Fever (3) Insomnia (1)	Low Ca (1) Low K (1) AST (1) High gluc (1)	Long QT (5) Tachycard (1) Orthostasis (1) Ging bleed (1) Oedema (1)
	3 and 4	Neutro (4) Anaemia (4) Platelets (7)	Diarrhoea (1)	Oedema (1)				Low Na (1)	MI (1) Neu fever (2)
1000/140 daily	1 and 2	Anaemia (3) Platelets (2)	Dry mouth (1) Nausea (2) Constip (1) Abd dist (1) Anorexia (1) Diarrhea (1)	Effusion (2)	Rash (1)	Headache (1) Dysaesthesia (1)	Fatigue (3) Chills (2) Hot flash (1) Wt loss (1) Insomnia (1) Sweats (1)	Alk phos (1) Low Na (1)	Long QT (1) Oedema (1) Weakness (1)
	3 and 4	Neutro (1) Platelets (1)		Dyspnea (2)					
1000/100 daily	1 and 2	Neutro (2) Anaemia (3) Platelets (3)	Nausea (6) Anorexia (2) Diarrhoea (2)	Dyspnea (1) Cough (1) Effusion (1)	Rash (4)	Headache (2) Abdomen (1)	Fatigue (6) Fever (1) Chills (1)	Alk phos (1) ALT (2) AST (1)	Epistaxis (1) Oedema (2)
	3 and 4	Neutro (5) Platelets (1)					Fatigue (1)		GI bleed (1)

Abbreviations: Abd dist=abdominal distention; Alb=albumin; Alk phos=alkaline phosphatase; ALT=alanine aminotransferase; AST=aspartate aminotransferase; Constip=constipation; Constit.=constitutional; Dehydr=dehydration; GI=gastrointestinal; gluc=glucose; HSR=hypersensitivity reaction; Integ.=integument; MI=myocardial infarction; Neutro=neutropenia; QT=QT interval in the electrocardiogram; Resp.=respiratory; Tongue sw=tongue swelling; Wt=weight.

Only the highest-grade toxicity for each patient is reported. Grade 3 and 4 toxicities are noted in shaded regions. The number of events is in parentheses.

**Table 3:** Individual patient DLTs (during DLT period, cycle 1, day 1–21) and proportion of patients with any grade 3 and 4 toxicity, possibly, probably, and definitely related to treatment

			**Patients with grade 3 or 4 toxicity by category (%)**						
**Cohort (dacarbazine/dasatinib)**	**DLT category (*N*)**	**DLT (%)**	**Heme**	**Pulm**	**Card**	**Bleed**	**Infect**	**GI**	**Cons**
1. 800/50 b.i.d.	—	0	—	—	—	—	—	—	—
2. 800/70 b.i.d.	—	0	9/19 (47)	1/19 (5)	—	—	—	—	—
3. 1000/70 b.i.d.	Heme (1) NF (2)^a^	21.4	10/14 (71)	1/14 (7)	1/14 (7)^b^	—	2/14 (14)^c^	1/14 (7)^d^	—
4. 1000/140 q.d.	Pulm (1) Heme (1)	33	1/3 (33)	2/3 (67)	—	—	—	—	—
5. 1000/100 q.d.	—	0	5/11 (45)	—	—	1/11 (9)^e^	—	—	1/11 (9)^f^
									
Total			25/50 (50)	3/50 (6)	1/50 (2)	1/50 (2)	2/50 (4)	1/50 (2)	1/50 (2)

Abbreviations: Bleed=bleeding; Card=cardiac; Cons=constitutional; DLT=dose-limiting toxicity; GI=gastrointestinal; Heme=hematologic; Infect=infection; NF=neutropenic fever; Pulm=pulmonary.

a Patients in expansion cohort.

b Myocardial infarction, possibly related.

c Febrile neutropenia.

d Diarrhoea.

e GI bleeding in the setting of thrombocytopenia.

f Fatigue.

**Table 4:** Best tumour response by RECIST criteria as a function of dacarbazine and dasatinib dose

**Dacarbazine (mg m^−2^**)	**Dasatinib**	**Not evaluable**	**PD (%)**	**SD (%)**	**PR (%)**	**CR**
800	50 mg b.i.d.	—	2/3	1/3	—	—
800	70 mg b.i.d.	1	5/18 (27.8)	11/18 (61.1)	2/18 (11.1)	—
1000	70 mg b.i.d.	3	3/11 (27.3)	6/11 (54.5)	2/11 (18.2)	—
1000	140 mg daily	—	1/3	2/3	—	—
1000	100 mg daily	—	5/11 (45.5)	6/11 (54.5)	—	—

Abbreviations: CR=complete response; PD=progressive disease; PR=partial response; RECIST=Response Evaluation Criteria in Solid Tumours; SD=stable disease.

Four patients were not evaluable for response due to adverse events before the first restaging scans.

## References

[bib1] Atkins MB, Kunkel L, Sznol M, Rosenberg SA (2000) High-dose recombinant interleukin-2 therapy in patients with metastatic melanoma: long-term survival update. Cancer J Sci Am 6(Suppl 1): S11–S14 10685652

[bib2] Atkins MB, Lotze MT, Dutcher JP, Fisher RI, Weiss G, Margolin K, Abrams J, Sznol M, Parkinson D, Hawkins M, Paradise C, Kunkel L, Rosenberg SA (1999) High-dose recombinant interleukin 2 therapy for patients with metastatic melanoma: analysis of 270 patients treated between 1985 and 1993. J Clin Oncol 17: 2105–2116 10561265 10.1200/JCO.1999.17.7.2105

[bib3] Barth A, Wanek LA, Morton DL (1995) Prognostic factors in 1521 melanoma patients with distant metastases. J Am Coll Surg 181: 193–201 7670677

[bib4] Beadling C, Jacobson-Dunlop E, Hodi FS, Le C, Warrick A, Patterson J, Town A, Harlow A, Cruz III F, Azar S, Rubin BP, Muller S, West R, Heinrich MC, Corless CL (2008) KIT gene mutations and copy number in melanoma subtypes. Clin Can Res 14: 6821–6828 10.1158/1078-0432.CCR-08-0575 18980976

[bib5] Boukerche H, Aissaoui H, Prévost C, Hirbec H, Das SK, Su Z, Sarkar D, Fisher PB (2010) Src kinase activation is mandatory for MDA-9/syntenin-mediated activation of nuclear factor-kappaB. Oncogene 29: 3054–3066 20228839 10.1038/onc.2010.65 PMC2878370

[bib6] Buettner R, Mesa T, Vultur A, Lee F, Jove R (2008) Inhibition of Src family kinases with dasatinib blocks migration and invasion of human melanoma cells. Mol Cancer Res 6: 1766–1774 19010823 10.1158/1541-7786.MCR-08-0169 PMC2768340

[bib7] Carvajal R, Chapman P, Wolchok J, Cane L, Teitcher J, Lutzky J, Pavlick A, Bastian BC, Antonescu CR, Schwartz GK (2009) A phase II study of imatinib mesylate (IM) for patients with advanced melanoma harboring somatic alterations of KIT. J Clin Oncol 27(Suppl 15s): abstr 9001

[bib8] Carvajal RD, Antonescu CR, Wolchok JD, Chapman PB, Roman R-A, Teitcher J, Panageas KS, Busam KJ, Chmielowski B, Lutzky J, Pavlick AC, Fusco A, Cane L, Takebe N, Vemula S, Bouvier N, Bastian BC, Schwartz GK (2011) KIT as a therapeutic target in metastatic melanoma. JAMA 305: 2327–2334 21642685 10.1001/jama.2011.746 PMC3986039

[bib9] Chapman PB, Einhorn LH, Meyers ML, Saxman S, Destro AN, Panageas KS, Begg CB, Agarwala SS, Schuchter LM, Ernstoff MS, Houghton AN, Kirkwood JM (1999) Phase III multicenter randomized trial of the Dartmouth regimen *vs* dacarbazine in patients with metastatic melanoma. J Clin Oncol 17: 2745–2751 10561349 10.1200/JCO.1999.17.9.2745

[bib10] Chapman PB, Hauschild A, Robert C, Haanen JB, Ascierto P, Larkin J, Dummer R, Garbe C, Testori A, Maio M, Hogg D, Lorigan P, Lebbe C, Jouary T, Schadendorf D, Ribas A, O’Day SJ, Sosman JA, Kirkwood JM, Eggermont AM, Dreno B, Nolop K, Li J, Nelson B, Hou J, Lee RJ, Flaherty KT, McArthur AG (2011) Improved survival with vemurafenib in melanoma with BRAF V600E mutation. N Engl J Med 364: 2507–2516 21639808 10.1056/NEJMoa1103782 PMC3549296

[bib11] ClinicalTrials.gov (no date) Dasatinib in Treating Patients With Locally Advanced or Metastatic Mucosal Melanoma, Acral Melanoma, or Solar Melanoma That Cannot Be Removed By Surgery – Full Text View. Retrieved 27 October 2010, from http://www.clinicaltrials.gov/ct2/show/NCT00700882?term=dasatinib+melanoma&rank=3

[bib12] Eustace A, Mahgoub T, Kennedy S, Crown J, Larkin A, Tryfonopoulos D, O’Driscoll L, Clynes M, O’Donovan N (2010) Targeting SRC kinase (SRC) in melanoma cells. J Clin Oncol 28(Suppl 15s): abstr 8584

[bib13] Eustace AJ, Crown J, Clynes M, O’Donovan N (2008) Preclinical evaluation of dasatinib, a potent Src kinase inhibitor, in melanoma cell lines. J Transl Med 6: 53 18823558 10.1186/1479-5876-6-53 PMC2569026

[bib14] Felicetti F, Parolini I, Bottero L, Fecchi K, Errico MC, Raggi C, Biffoni M, Spadaro F, Lisanti MP, Sargiacomo M, Carè A (2009) Caveolin-1 tumor-promoting role in human melanoma. Int J Cancer 125: 1514–1522 19521982 10.1002/ijc.24451 PMC2805039

[bib15] Finn RS, Dering J, Ginther C, Wilson CA, Glaspy P, Tchekmedyian N, Slamon DJ (2007) Dasatinib, an orally active small molecule inhibitor of both the src and abl kinases, selectively inhibits growth of basal-type/‘triple-negative’ breast cancer cell lines growing *in vitro*. Breast Cancer Res Treat 105: 319–326 17268817 10.1007/s10549-006-9463-x

[bib16] Flaherty KT, Puzanov I, Kim KB, Ribas A, McArthur GA, Sosman JA, O’Dwyer PJ, Lee RJ, Grippo JF, Nolop K, Chapman PB (2010) Inhibition of mutated, activated BRAF in metastatic melanoma. N Engl J Med 363: 809–819 20818844 10.1056/NEJMoa1002011 PMC3724529

[bib17] Fraser CK, Lousberg EL, Guerin LR, Hughes TP, Brown MP, Diener KR, Hayball JD (2010) Dasatinib alters the metastatic phenotype of B16-OVA melanoma *in vivo*. Cancer Biol Ther 10: 715–727. Retrieved from http://www.ncbi.nlm.nih.gov/pubmed/20676039 20676039 10.4161/cbt.10.7.12926

[bib18] Guo J, Si L, Kong Y, Flaherty KT, Xu X, Zhu Y, Corless CL, Li L, Li H, Sheng X, Cui C, Chi Z, Li S, Han M, Mao L, Lin X, Du N, Zhang X, Li J, Wang B, Qin S (2011) Phase II, open-label, single-arm trial of imatinib mesylate in patients with metastatic melanoma harboring c-Kit mutation or amplification. J Clin Oncol 29: 2904–2909 21690468 10.1200/JCO.2010.33.9275

[bib19] Handolias D, Hamilton AL, Salemi R, Tan A, Moodie K, Kerr L, Dobrovic A, McArthur GA (2010) Clinical responses observed with imatinib or sorafenib in melanoma patients expressing mutations in KIT. Br J Cancer 102: 1219–1223 20372153 10.1038/sj.bjc.6605635 PMC2856012

[bib20] Hiscox S, Morgan L, Green TP, Barrow D, Gee J, Nicholson RI (2006) Elevated Src activity promotes cellular invasion and motility in tamoxifen resistant breast cancer cells. Breast Cancer Res Treat 97: 263–274 16333527 10.1007/s10549-005-9120-9

[bib21] Hodi FS, O’Day SJ, McDermott DF, Weber RW, Sosman JA, Haanen JB, Gonzalez R, Robert C, Schadendorf D, Hassel JC, Akerley W, van den Eertwegh AJ, Lutzky J, Lorigan P, Vaubel JM, Linette GP, Hogg D, Ottensmeier CH, Lebbé C, Peschel C, Quirt I, Clark JI, Wolchok JD, Weber JS, Tian J, Yellin MJ, Nichol GM, Hoos A, Urba WJ (2010) Improved survival with ipilimumab in patients with metastatic melanoma. N Engl J Med 363: 711–723 20525992 10.1056/NEJMoa1003466 PMC3549297

[bib22] Homsi J, Cubitt CL, Zhang S, Munster PN, Yu H, Sullivan DM, Jove R, Messina JL, Daud AI (2009) Src activation in melanoma and Src inhibitors as therapeutic agents in melanoma. Melanoma Res 19: 167–175 19434004 10.1097/CMR.0b013e328304974c

[bib23] Huang F, Reeves K, Han X, Fairchild C, Platero S, Wong TW, Lee F, Shaw P, Clark E (2007) Identification of candidate molecular markers predicting sensitivity in solid tumors to dasatinib: rationale for patient selection. Cancer Res 67: 2226–2238 17332353 10.1158/0008-5472.CAN-06-3633

[bib24] Irby RB, Malek RL, Bloom G, Tsai J, Letwin N, Frank BC, Verratti K, Yeatman TJ, Lee NH (2005) Iterative microarray and RNA interference-based interrogation of the SRC-induced invasive phenotype. Cancer Res 65: 1814–1821 15753379 10.1158/0008-5472.CAN-04-3609

[bib25] Irby RB, Mao W, Coppola D, Kang J, Loubeau JM, Trudeau W, Karl R, Fujita DJ, Jove R, Yeatman TJ (1999) Activating SRC mutation in a subset of advanced human colon cancers. Nat Genet 21: 187–190 9988270 10.1038/5971

[bib26] Jallal H, Valentino M, Chen G, Boschelli F, Ali S, Rabbani SA (2007) A Src/Abl kinase inhibitor, SKI-606, blocks breast cancer invasion, growth, and metastasis *in vitro* and *in vivo*. Cancer Res 67: 1580–1588 17308097 10.1158/0008-5472.CAN-06-2027

[bib27] Jemal A, Siegel R, Xu J, Ward E (2010) Cancer Statistics, 2010. CA Cancer J Clin 60: 277–300 20610543 10.3322/caac.20073

[bib28] Jilaveanu LB, Zito CR, Aziz SA, Chakraborty A, Davies MA, Camp RL, Rimm DL, Dudek A, Sznol M, Kluger HM (2011) *In vitro* studies of dasatinib, its targets and predictors of sensitivity. Pigment Cell Melanoma Res 24: 386–389 21320292 10.1111/j.1755-148X.2011.00835.x PMC4431976

[bib29] Journe F, Wiedig M, Morandini R, Sales F, Ghanem G, Awada A (2010) cKIT overexpression and wild-type NRAS/BRAF predict response to the tyrosine kinase inhibitor dasatinib in melanoma cell lines. Eur J Cancer 8: 83–84, abstract 254

[bib30] Kefford R, Arkenau H, Brown M, Millward M, Infante J, Long G, Ouellet D, Curtis M, Lebowitz PF, Falchook GS (2010) Phase I/II study of GSK2118436, a selective inhibitor of oncogenic mutant BRAF kinase, in patients with metastatic melanoma and other solid tumors. J ClinOncol 28(Suppl 15s): abstr 8503

[bib31] Kim LC, Song L, Haura EB (2009) Src kinases as therapeutic targets for cancer. Nat Rev Clin Oncol 6: 587–595 19787002 10.1038/nrclinonc.2009.129

[bib32] Kline CLB, Jackson R, Engelman R, Pledger WJ, Yeatman TJ, Irby RB (2008) Src kinase induces tumor formation in the c-SRC C57BL/6 mouse. Int J Cancer 122: 2665–2673 18351644 10.1002/ijc.23445

[bib33] Kluger HM, Dudek AZ, McCann C, Ritacco J, Southard N, Jilaveanu LB, Molinaro A, Sznol M (2011) A phase 2 trial of dasatinib in advanced melanoma. Cancer 117: 2202–2208 21523734 10.1002/cncr.25766 PMC3116034

[bib34] Korn EL, Liu P, Lee SJ, Chapman JW, Niedzwiecki D, Suman VJ, Moon J, Sondak VK, Atkins MB, Eisenhauer EA, Parulekar W, Markovic SN, Saxman S, Kirkwood JM (2008) Meta-analysis of phase II cooperative group trials in metastatic stage IV melanoma to determine progression-free and overall survival benchmarks for future Phase II trials. J Clin Oncol 26: 527–534 18235113 10.1200/JCO.2007.12.7837

[bib35] Kotha A, Sekharam M, Cilenti L, Siddiquee K, Khaled A, Zervos AS, Carter B, Turkson J, Jove R (2006) Resveratrol inhibits Src and Stat3 signaling and induces the apoptosis of malignant cells containing activated Stat3 protein. Mol Cancer Ther 5: 621–629 16546976 10.1158/1535-7163.MCT-05-0268

[bib36] Luikart SD, Kennealey GT, Kirkwood JM (1984) Randomized phase III trial of vinblastine, bleomycin, and cis-dichlorodiammine-platinum *vs* dacarbazine in malignant melanoma. J Clin Oncol 2: 164–168 6199481 10.1200/JCO.1984.2.3.164

[bib37] Lutzky J, Bauer J, Bastian BC (2008) Dose-dependent, complete response to imatinib of a metastatic mucosal melanoma with a K642E KIT mutation. Pigment Cell Melanoma Res 21: 492–493 18510589 10.1111/j.1755-148X.2008.00475.x

[bib38] Maat W, el Filali M, Dirks-Mulder A, Luyten GPM, Gruis NA, Desjardins L, Boender P, Jager MJ, van der Velden PA (2009) Episodic Src activation in uveal melanoma revealed by kinase activity profiling. Br J Cancer 101: 312–319 19568237 10.1038/sj.bjc.6605172 PMC2720193

[bib39] Middleton MR, Grob JJ, Aaronson N, Fierlbeck G, Tilgen W, Seiter S, Gore M, Aamdal S, Cebon J, Coates A, Dreno B, Henz M, Schadendorf D, Kapp A, Weiss J, Fraass U, Statkevich P, Muller M, Thatcher N (2000) Randomized phase III study of temozolomide *vs* dacarbazine in the treatment of patients with advanced metastatic malignant melanoma. J Clin Oncol 18: 158–166 10623706 10.1200/JCO.2000.18.1.158

[bib40] Morgan L, Gee J, Pumford S, Farrow L, Finlay P, Robertson J, Ellis I, Kawakatsu H, Nicholson R, Hiscox S (2009) Elevated Src kinase activity attenuates Tamoxifen response *in vitro* and is associated with poor prognosis clinically. Cancer Biol Ther 8: 1550–1558 19830888 10.4161/cbt.8.16.8954

[bib41] Nam S, Kim D, Cheng JQ, Zhang S, Lee J, Buettner R, Mirosevich J, Lee FY, Jove R (2005) Action of the Src family kinase inhibitor, dasatinib (BMS-354825), on human prostate cancer cells. Cancer Res 65: 9185–9189 16230377 10.1158/0008-5472.CAN-05-1731

[bib42] Niu G, Bowman T, Huang M, Shivers S, Reintgen D, Daud A, Chang A, Kraker A, Jove R, Yu H (2002) Roles of activated Src and Stat3 signaling in melanoma tumor cell growth. Oncogene 21: 7001–7010 12370822 10.1038/sj.onc.1205859

[bib43] Ratain MJ, Mick R, Schilsky RL, Siegler M (1993) Statistical and ethical issues in the design and conduct of phase I and II clinical trials of new anticancer agents. J Natl Cancer Inst 85: 1637–1643 8411243 10.1093/jnci/85.20.1637

[bib44] Robert C, Thomas L, Bondarenko I, O’Day S, Weber J, Garbe C, Lebbe C, Baurain JF, Testori A, Grob JJ, Davidson N, Richards J, Maio M, Hauschild A, Miller Jr WH, Gascon P, Lotem M, Harmankaya K, Ibrahim R, Francis S, Chen TT, Humphrey R, Hoos A, Wolchok JD (2011) Ipilimumab plus dacarbazine for previously untreated metastatic melanoma. N Engl J Med 364: 2517–2526 21639810 10.1056/NEJMoa1104621

[bib45] Satzger I, Schaefer T, Kuettler U, Broecker V, Voelker B, Ostertag H, Kapp A, Gutzmer R (2008) Analysis of c-KIT expression and KIT gene mutation in human mucosal melanomas. Br J Cancer 99: 2065–2069 19018266 10.1038/sj.bjc.6604791 PMC2607233

[bib46] Schadendorf D, Ugurel S, Schuler-Thurner B, Nestle FO, Enk A, Bröcker E, Grabbe S, Rittgen W, Edler L, Sucker A, Zimpfer-Rechner C, Berger T, Kamarashev J, Burg G, Jonuleit H, Tüttenberg A, Becker JC, Keikavoussi P, Kämpgen E, Schuler G (2006) Dacarbazine (DTIC) *vs* vaccination with autologous peptide-pulsed dendritic cells (DC) in first-line treatment of patients with metastatic melanoma: a randomized phase III trial of the DC study group of the DeCOG. Ann Oncol 17: 563–570 16418308 10.1093/annonc/mdj138

[bib47] Song L, Morris M, Bagui T, Lee FY, Jove R, Haura EB (2006) Dasatinib (BMS-354825) selectively induces apoptosis in lung cancer cells dependent on epidermal growth factor receptor signaling for survival. Cancer Res 66: 5542–5548 16740687 10.1158/0008-5472.CAN-05-4620

[bib48] Torres-Cabala CA, Wang W, Trent J, Yang D, Chen S, Galbincea J, Kim KB, Woodman S, Davies M, Plaza JA, Nash JW, Prieto VG, Lazar AJ, Ivan D (2009) Correlation between KIT expression and KIT mutation in melanoma: a study of 173 cases with emphasis on the acral-lentiginous/mucosal type. Mod Pathol 22: 1446–1456 19718013 10.1038/modpathol.2009.116 PMC4120323

[bib49] Trevino JG, Summy JM, Lesslie DP, Parikh NU, Hong DS, Lee FY, Donato NJ, Abbruzzese JL, Baker CH, Gallick GE (2006) Inhibition of SRC expression and activity inhibits tumor progression and metastasis of human pancreatic adenocarcinoma cells in an orthotopic nude mouse model. Am J Pathol 168: 962–972 16507911 10.2353/ajpath.2006.050570 PMC1606527

[bib50] Trimmer C, Whitaker-Menezes D, Bonuccelli G, Milliman JN, Daumer KM, Aplin AE, Pestell RG, Sotgia F, Lisanti MP, Capozza F (2010) CAV1 inhibits metastatic potential in melanomas through suppression of the integrin/Src/FAK signaling pathway. Cancer Res 70: 7489–7499 20709760 10.1158/0008-5472.CAN-10-0900 PMC2948597

[bib51] Wang X, Reeves K, Luo FR, Xu L, Lee F, Clark E, Huang F (2007) Identification of candidate predictive and surrogate molecular markers for dasatinib in prostate cancer: rationale for patient selection and efficacy monitoring. Genome Biol 8: R255 18047674 10.1186/gb-2007-8-11-r255 PMC2258199

[bib52] Woodman SE, Trent JC, Stemke-Hale K, Lazar AJ, Pricl S, Pavan GM, Fermeglia M, Gopal YN, Yang D, Podoloff DA, Ivan D, Kim KB, Papadopoulos N, Hwu P, Mills GB, Davies MA (2009) Activity of dasatinib against L576P KIT mutant melanoma: molecular, cellular, and clinical correlates. Mol Cancer Ther 8: 2079–2085 19671763 10.1158/1535-7163.MCT-09-0459 PMC3346953

[bib53] Yu CL, Meyer DJ, Campbell GS, Larner AC, Carter-Su C, Schwartz J, Jove R (1995) Enhanced DNA-binding activity of a Stat3-related protein in cells transformed by the Src oncoprotein. Science 269: 81–83 7541555 10.1126/science.7541555

